# The association between lunar phase and intracranial aneurysm rupture: Myth or reality? Own data and systematic review

**DOI:** 10.1186/s12883-017-0879-1

**Published:** 2017-05-19

**Authors:** Adomas Bunevicius, Agne Gendvilaite, Vytenis Pranas Deltuva, Arimantas Tamasauskas

**Affiliations:** 1 Institute of Neurosciences, Lithuanian University of Health Sciences, Eiveniu g. 2, LT-50009 Kaunas, Lithuania; 2 Department of Neurosurgery, Hospital of Lithuanian University of Health Sciences Kaunas Clinics, Kaunas, Lithuania; 3 Faculty of Medicine, Lithuanian University of Health Sciences, Kaunas, Lithuania

## Abstract

**Background:**

It is a common belief in medical community that lunar phases have an impact on human health. A growing body of evidence indicates that lunar phases can predict the risk to develop acute neurological and vascular disorders. The goal of present report was to present our institution data and to perform systematic review of studies examining the association of intracranial aneurysm rupture with moon phases.

**Methods:**

We identified all patients admitted to our department for ruptured intracranial aneurysms in a period between November, 2011 and December, 2014. Patients with a known aneurysm rupture date were included. Lunar phases were determined by dividing lunar month (29.5 days) into eight equal parts, i.e., new moon, waxing crescent, first quarter, waxing gibbous, full moon, waning gibbous, last quarter and waning crescent. A systematic literature review was undertaken to identify studies that evaluated the association of lunar phases with the incident of intracranial aneurysm rupture.

**Result:**

One hundred and eighty-six patients (62 men and 124 women, median age 56 years) were admitted to our department for treatment of ruptured intracranial aneurysms. The rate of intracranial aneurysm rupture was equally distributed across all phases of the lunar cycle (*X* ^*2*^ [7; 185] = 12.280, *p* = 0.092). We identified three studies that evaluated the association between incident intracranial aneurysm rupture and lunar phases with a total of 1483 patients. One study from Lebanon found that the incidence rate of intracranial aneurysm rupture was statistically significantly greater during the new moon phase (25% cases), relative to the other seven lunar phases (*p* < 0.001). Two subsequent studies from Austria and Germany in larger patient samples (*n* = 717 and *n* = 655, respectively) did not find an association between lunar phases and intracranial aneurysm rupture (*p*-values of 0.84 and 0.97, respectively). When analyzing all four studies together, we did not find an association between lunar phases and incidence of intracranial aneurysm rupture (*X* ^*2*^ [1668; 7] = 2.080, *p* = 0.955).

**Conclusions:**

Moon phases are not associated with incidence of intracranial aneurysm rupture. Studies investigating the association of intracranial aneurysm rupture with lunar illumination defined using more sensitive approaches are encouraged.

## Background

Five percent of the normal adult population harbor an intracranial aneurysm which can be potentially lethal in case of rupture [1]. Rupture is the most common initial presentation of intracranial aneurysms and it is the third commonest type of stroke. Ruptured brain aneurysms are fatal in up to 40% of cases and one third of survivors suffer from permanent neurological deficits [1, 2]. Clinical risk factors of intracranial aneurysm rupture include increasing age, arterial hypertension, atherosclerosis, greater aneurysm size, smoking and heavy alcohol consumption [3–5]. Circadian and seasonal rhythmic variations are associated with vascular functioning [6] and were implicated in the risk to develop acute cerebrovascular events [7, 8]. For example, cold weather, epidemic influenza [9] and spring season [10] were linked to greater risk of intracranial aneurysm rupture. On the other hand, others reported that low temperature and high barometric pressure may be risk factors for aneurysm rupture [11].

In a medical community it is commonly believed that moon has an effect on human health [12]. An increasing body of evidence suggests that lunar phases can be associated with the incidence of acute cerebrovascular events. It was shown that full moon is associated with greater incidence of hemorrhagic stroke, transient ischemic attack [8] and unexplained stroke symptoms [13] but not with ischemic stroke. There is a common belief, which is based mostly on anecdotal experiences, that the waxing phase of the moon can be associated with greater surgical complication risk [14] and that the incidence of intracranial aneurysm rupture increases during the new moon phase [15]. Therefore, the goal of present report was to present our institution data and to perform systematic review of studies examining the association of intracranial aneurysm rupture with moon phases.

## Methods

### Patients

We retrospectively identified all patients admitted to our department in a period from November, 2011 until December, 2014 with spontaneous subarachnoid hemorrhage (SAH) diagnosed using non-contrast head CT due to intracranial aneurysm rupture verified using CT angiography or digital subtraction angiography. Patients were excluded if they were diagnosed with non-ruptured intracranial aneurysms or traumatic SAH, or with uncertain SAH onset. Time of aneurysm rupture was documented as anamnestic onset of headache or neurological symptoms as documented in medical records. Charts were reviewed for patients’ age, gender, admission Glasgow Coma scale score, Hunt-Hess grade, World Federation of Neurosurgical Societies grade and bleeding site.

### Lunar cycle assessment

Determination of the lunar cycle was based on previous studies [15–17]. The lunar cycle (or month) includes 29.5 days. We used the United States Naval Observatory online website (www.usno.navy.mil/USNO) to determine the beginning of the new moon. Subsequently, the lunar month was divided into eight equal parts, i.e., new moon, waxing crescent, first quarter, waxing gibbous, full moon, waning gibbous, last quarter and waning crescent. Time of aneurysm rupture was assigned to the particular lunar phase.

### Systematic review

A systematic literature review was conducted on December 25, 2016 to identify studies that evaluated the association of lunar phases with the incident of intracranial aneurysm rupture. Articles for review were identified from the PubMed by using the following key-words: “lunar phase”, “moon phase”, “subarachnoid hemorrhage”, “aneurysm rupture”, “intracranial aneurysm” and “cerebral aneurysm”. There were no restrictions regarding the year of publication; however, only papers with their abstract or full-text written in English were considered. Review papers were excluded from the analysis. Identified papers were also reviewed for other relevant studies.

An initial literature search was performed by reviewing titles and abstracts of papers, and relevant full-text articles were extracted for final analyses. Full-text articles of the selected studies were reviewed for study year, country of publication, study inclusion criteria, sample size, definition of lunar phases and aneurysmal SAH, and incidence of aneurysmal SAH across the lunar phases.

### Statistical analyses

Statistical analyses were performed using the IBM SPSS Version 19 software. Categorical variables are presented as count (percentage) and continuous data as median (minimal value – maximal value). Chi-squared goodness-of-fit was calculated to determine differences of the incident of aneurysm rupture across eight lunar phase cycles. A *p* value of 0.05 or less was considered as significant.

## Results

During the study period a total of 186 patients (62 men and 124 women, median age 56 years) were admitted to our department for treatment of ruptured intracranial aneurysms and with known rupture date (Table 1). The most common bleeding sites were anterior communicating artery (30%), internal carotid artery (25%) and medial cerebral artery (20%). The rate of intracranial aneurysm rupture was equally distributed across all phases of the lunar cycle (*X* ^*2*^ [7; 185] = 12.280, *p* = 0.092; Table 2).

**Table 1:** Clinical and demographic characteristics of the study patients (*n* = 186)

Characteristic	Median (min - max) or number (percent)
**Age (years)**	
Median (min – max)	56 (25–93)
**Gender, n (%)**	
Men	62 (33%)
Women	124 (67%)
**Glasgow Coma score**	
Median (min – max)	15 (4–15)
**Hunt-Hess grade**	
Median (min – max)	1 (1–5)
**World Federation of Neurosurgical Societies grading system**	
Median (min – max)	1 (1–5)
**Bleeding site**	
Anterior communicating artery	56 (30%)
Internal carotid artery	47 (25%)
Medial cerebral artery	38 (20%)
Anterior cerebral artery	16 (9%)
Basilar artery	8 (4%)
Vertebral artery	3 (2%)
Multiple aneurysms	5 (3%)
Other	13 (7%)

**Table 2:**
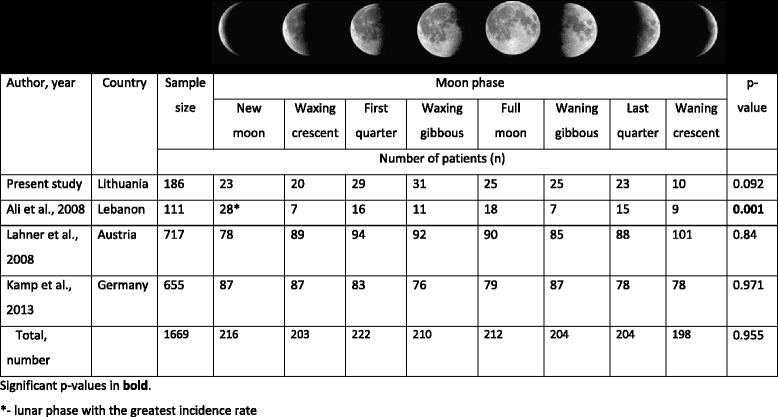
Association between lunar phase and intracranial aneurysm rupture

Results of systematic review are presented in Table 1. We identified three studies that evaluated the association between the incident of intracranial aneurysm rupture and lunar phases. A total of 1483 patients were included across the three studies with sample sizes across individual studies ranging between 111 [15] and 717 [17] patients. A study from Lebanon by Ali and colleagues found that the incidence rate of intracranial aneurysm rupture was statistically significantly greater during the new moon phase (25% of cases), relative to the other seven lunar phases (*p* < 0.001) [15]. Two subsequent studies from Austria [17] and Germany [16] in larger patient samples (*n* = 717 and *n* = 655, respectively) did not find an association between lunar phases and intracranial aneurysm rupture (*p* values of 0.84 and 0.97, respectively). When analyzing all four studies together, we did not find an association between the lunar phases and incidence of intracranial aneurysm rupture (*X* ^*2*^ [1668; 7] = 2.080, *p* = 0.955; Table 2).

## Discussion

Our experience and systematic review indicate that the incidence of intracranial aneurysm rupture is not associated with lunar phases.

In our cohort, lunar phases were not associated with the incidence rate of intracranial aneurysm rupture. We identified three previous studies from Lebanon [15], Austria [17] and Germany [16] that looked into the potential association of intracranial aneurysm rupture with lunar phases. A study from the Lebanon have reported that the incidence rate of intracranial aneurysm rupture was the greatest during the new moon phase, relative to the other lunar phases [15]. However, the study results should be interpreted with caution due to small sample size (*n* = 111). Subsequent substantially larger studies from Austria (*n* = 717) and Germany (*n* = 655) did not find an association between lunar phases and the incidence of intracranial aneurysm rupture. When we analyzed the results of the four studies with a total of 1669 patients together, we did not find an association between intracranial aneurysm rupture and lunar phases. These findings indicate that lunar phases are not associated with the incidence of intracranial aneurysm rupture.

Methodological implications should be considered when interpreting our findings. Specifically, in ours and three reviewed studies the lunar cycle phases were determined by dividing the lunar month into eight equal parts of 3.7 days each based on the date of the new moon. While such an approach is familiar and appealing, but it prevents from using more sensitive analyses when evaluating the impact of lunar illumination on health. In a recent retrospective study from the United States, Banfield with colleagues evaluated the association of intracranial aneurysm rupture with the degree of lunar illumination divided into 5 categories over a 10-year period, and found that odds for intracranial aneurysm rupture was the greatest when the moon was the least (new moon) and the most (full moon) illuminated when comparing to the middle of the lunar cycle [18]. However, it should be noted that the latter study included only patients admitted for endovascular coiling of ruptured intracranial aneurysms at a single institution. Nevertheless, future studies should attempt to evaluate the association of the lunar illumination degree with the incidence of aneurysm rupture.

Strong theoretical models explaining the impact of moon on human health and functioning of cardiovascular system are lacking, therefore resulting in a substantial degree of skepticism regarding such an association [17, 19, 20]. However, others believe that health implications of moonlight chronobiology remain poorly understood and should be an important research topic [21]. Furthermore, it is difficult to ignore a common belief among medical professionals that moon has an impact on human health [12]. Substantial evidence suggests that lunar phases can be associated with the incidence of acute neurological and vascular disorders, including hemorrhagic stroke [8], gastrointestinal bleeding, [22], seizures [23] and myocardial infarction [24]. However, others did not find an association of moon phases with the incidence of seizures [25] and ischemic stroke [8]. Moon may also impact other bodily functions, such as blood pressure and sleep [26] that can subsequently predispose to intracranial aneurysm rupture. We believe that the potential impact of moon on human health should be studied in the future; however, methodologically and statistically rigorous approaches should be employed and results should be interpreted with caution. Other more important risk factors for intracranial aneurysm development and rupture should receive more attention from researchers and clinicians.

The study has limitations. Our results are at risk for selection bias because it was performed at in-hospital setting and included a single institution experience. Retrospective design is another limitation of the study that prevented from more accurate identification of timing of aneurysm rupture and other behavioral (e.g., smoking) and cardiovascular (e.g., arterial hypertension, blood pressure preceding or immediately after aneurysm rupture) risk factors for intracranial aneurysm development and rupture. Moderate sample size of our cohort limits statistical power of the analysis. However, systematic review allowed us to substantially increase the statistical power.

## Conclusions

Our institution findings and systematic review indicate that moon phases are not associated with the incidence of intracranial aneurysm rupture. Studies investigating the association of intracranial aneurysm rupture with lunar illumination defined using more sensitive approaches are encouraged.

## Abbreviations

CT: computed tomography
SAH: subarachnoid hemorrhage
